# The pressure sensitivity of wrinkled B-doped nanocrystalline diamond membranes

**DOI:** 10.1038/srep35667

**Published:** 2016-10-21

**Authors:** S. Drijkoningen, S. D. Janssens, P. Pobedinskas, S. Koizumi, M. K. Van Bael, K. Haenen

**Affiliations:** 1 Institute for Materials Research (IMO), Hasselt University, Diepenbeek, Belgium; 2 IMOMEC, IMEC vzw, Diepenbeek, Belgium; 3 National Institute for Materials Science (NIMS), Tsukuba, Ibaraki, Japan; 4 Core Research for Evolutional Science and Technology (CREST), Japan Science and Technology Agency (JST), c/o AIST, Tsukuba, Ibaraki, Japan

## Abstract

Nanocrystalline diamond (NCD) membranes are promising candidates for use as sensitive pressure sensors. NCD membranes are able to withstand harsh conditions and are easily fabricated on glass. In this study the sensitivity of heavily boron doped NCD (B:NCD) pressure sensors is evaluated with respect to different types of supporting glass substrates, doping levels and membrane sizes. Higher pressure sensing sensitivities are obtained for membranes on Corning Eagle 2000 glass, which have a better match in thermal expansion coefficient with diamond compared to those on Schott AF45 glass. In addition, it is shown that larger and more heavily doped membranes are more sensitive. After fabrication of the membranes, the stress in the B:NCD films is released by the emergence of wrinkles. A better match between the thermal expansion coefficient of the NCD layer and the underlying substrate results in less stress and a smaller amount of wrinkles as confirmed by Raman spectroscopy and 3D surface imaging.

The fabrication and application of nanocrystalline diamond (NCD) membranes is an interesting and developing research topic. Kummer *et al*. showed that thin conductive diamond films can be used for *in-situ* monitoring of the intensity of soft X-ray beams^1^. Williams *et al*. demonstrated tuneable lenses out of boron doped NCD (B:NCD) films^2^. Even 300 nm thick single crystal diamond membranes were reported for use as optical resonators for colour centers^3^. Additionally, research of pressure sensors based on piezoresistive materials has been carried out extensively and is well described in literature^4^. Piezoresistive sensors are being used for industrial and for biomedical applications, e.g. for the measurement of blood pressure^5^. The gauge factor, a measure for the relative change in electrical resistance compared to the applied mechanical strain, depends on the nature of geometrical properties of the piezoresistive material. In order to maximise the sensitivity of pressure sensors we need to increase the gauge factor as much as possible. NCD diamond thin films typically have gauge factors of up to 30, which is comparable to that of silicon^6^. Whereas commercial silicon pressure sensors are still widely used, diamond offers important benefits in applications where silicon cuts short. Diamond is corrosion resistant which means no additional passivation layer is required to use the sensors in corrosive, electrochemical or harsh media. In addition, the radiation hardness, thermal conductivity and biocompatibility are far superior for diamond.

The current state-of-the-art piezoresistive sensors are mostly based on a non-piezoresistive membrane or diaphragm to which piezoresistive components are attached. For instance, piezoresistive boron-doped nanocrystalline diamond (B:NCD) films or structures have been attached to Si diaphragms^7^,^8^. The direct construction of membranes out of piezoresistive material, however, reduces the number of fabrication steps, e.g. diamond membranes of a few micrometres thick have been demonstrated^8^,^9^,^10^. Due to the high Young´s modulus and fracture strength of diamond much thinner films can be fabricated compared to other materials, which means that less pressure is required for a significant response, i.e. a higher value for the gauge factor is obtained. It was shown that B:NCD membranes of only few hundred nanometres thick can be used as sensitive pressure sensors^11^. Moreover, the underlying substrate affects the properties of the resulting diamond film since the amount of stress in the film is highly dependent on the differences in thermal expansion coefficients between the diamond and the substrate. To investigate the influence of the size of the membranes, the boron doping level and of the substrate material, and - more specifically of its thermal expansion coefficient, on the gauge factor and thus sensitivity of the resulting B:NCD membranes, films with different doping concentrations were grown onto two different types of borosilicate glass, Schott AF45 (S-AF45) and Corning Eagle 2000 (CE2000), of which the latter has a higher glass transition temperature. Additional characterization was done by scanning electron microscopy (SEM), Raman spectroscopy and laser scanning microscopy; the results were compared with respect to the sensitivity of the membranes. The difference in thermal expansion coefficient causes a difference in stress in the diamond films upon cooling after growth, which is directly observable by the number of wrinkles present in the membranes and which influences their sensitivity to pressure differences. Laser scanning micrographs confirm that a higher number of wrinkles is present for glass with a higher expansion coefficient (S-AF45) and reveal that the first generation wrinkles transform into the next generation closer to the membrane edge compared to glass with a lower expansion coefficient (CE2000).

## Results and Discussion

### Piezoresistive effect

**Figure 1:**
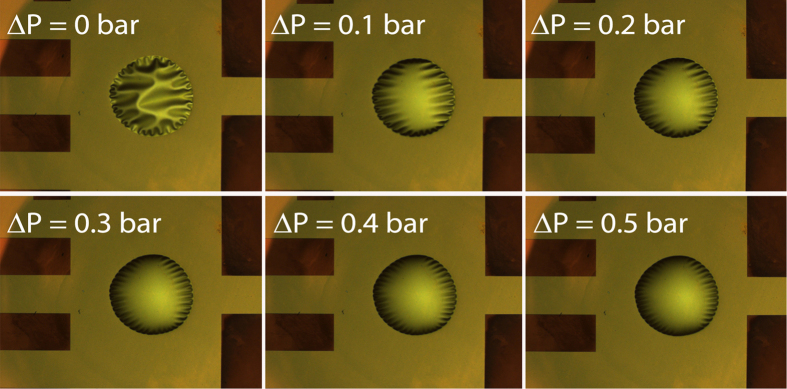
Optical images of an NCD membrane on CE2000 glass with 560 μm diameter upon increase of the applied differential pressure. From top left to bottom right the differential pressure is 0, 0.1, 0.2, 0.3, 0. 4, 0.5 bar respectively.

In order to produce pressure sensors that can compete with commercially available silicon sensors, it is important to reach high sensitivities. For this study, 3 types of membranes were prepared on 2 types of glass (S-AF45 and CE2000) to illustrate their typical behaviour. The relative change in resistance of the samples is determined as function of applied differential pressure. An increase of the applied differential pressure causes bulging and stretching of the NCD membrane (Fig. 1). A maximum differential pressure of 0.8 bar was applied to ensure that no damage is caused to the membranes. After local removal of the substrate, the membranes are clearly wrinkled. The wrinkles become less pronounced upon bulging of the membrane. After deflation the original wrinkle pattern reappears entirely both around the circumference and in the central part.

**Figure 2:**
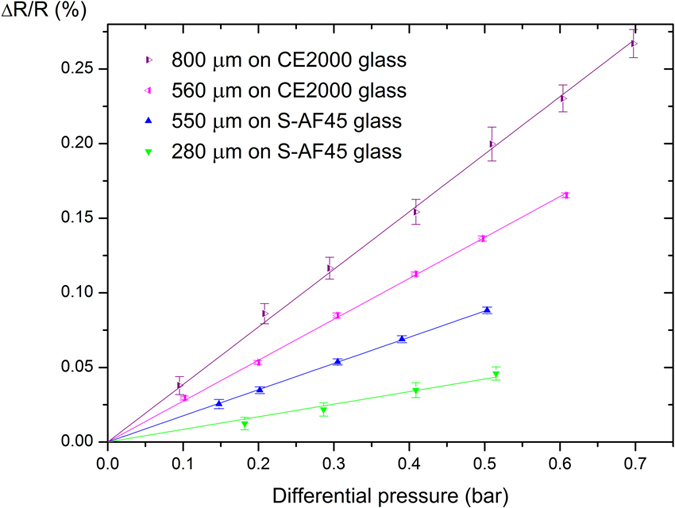
Relative change in resistance as a function of applied differential pressure for a 280 μm diameter membrane (green triangles) and a 550 μm diameter membrane (blue triangles) on S-AF45 glass and for a 560 μm diameter membrane (pink triangles) and a 800 μm diameter membrane (purple triangles) on CE 2000 glass.

As expected, based on the piezoresistive effect, the resistance increases upon bulging of the membrane (Fig. 2) 11. For hydrogen terminated nanocrystalline diamond (NCD:H) the piezoresistive increase is reasoned to originate directly from strain-induced changes in the resistivity of the grain boundaries^12^, for B:NCD membranes however the influence of the grain boundaries on this increase in resistance remains unclear. Turner *et al*. showed that boron dopant atoms are incorporated to the same extent both in the diamond grains and in the grain boundaries^13^. In the grains, boron is incorporated substitutionally within the diamond lattice, while in the grain boundaries boron atoms are surrounded with amorphous carbon. What the exact influence of both types of boron incorporation on the conductivity and piezoresistivity of B:NCD is, remains a question, although Janssens *et al*. already showed that very small variations in boron incorporation and number of grain boundaries are detectable in electric transport measurements^14^. In order to obtain a universal method of quantification for the piezoresistive effect, the pressure sensitivity *S*, which is defined as


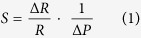


with *R* the resistance and *ΔP* the change in differential pressure, was determined^15^. The resistivity of the samples depend on the measurement conditions, e.g. state of the contacts, and is in the range of 1 to 17 kΩ for our samples. The obtained values for the relative change in resistivity were measured multiple times over a period of a month and have proven to be reproducible, although the absolute resistance varies with the state of the contacts and other factors like exact grain structure and doping level. It is exactly because of this variation in absolute resistance, when multiple samples are compared that the relative resistance was measured, which is a reproducible quantity. For the 4 membranes studied in Fig. 2, the estimated boron content, as determined from Raman spectroscopy is 8·10^21 ^cm^−3^, a value well over the Mott transition, rendering these films effectively into semi-metallic conductors^16^.

### Substrate dependence of the sensitivity

It is extremely difficult to produce membranes with the exact same diameter because the etch rate of the glasses are only estimates, influenced by local impurities, and once the diamond layer is reached, the membrane radius increases very fast. To assess the influence of the substrate on the sensitivity of the membrane, two membranes with comparable size, 550 μm diameter and 560 μm diameter on S-AF45 and CE 2000 glass, respectively, were fabricated and the relative change in resistance was compared (Fig. 2).

With a change of the glass substrate from S-AF45 to CE2000, the sensitivity increases with 55%. This increase is attributed to the difference in stress in the resulting film caused by the aforementioned difference in thermal expansion coefficient of the two types of glass with respect to the thermal expansion coefficient of the diamond film. Wur and Davidson reported a similar dependence of the piezoresistivity of microcrystalline diamond films on the host substrate^17^. Dependent on whether the thermal expansion coefficient of the underlying substrate is smaller or larger than that of diamond (1 × 10^−6^ °C^−1^) tensile or compressive stress, respectively, will build up in the NCD film. The thermal expansion coefficients of S-AF45 glass is higher than that of CE2000 glass which leads to a higher amount of compressive stress in NCD layers grown on S-AF45 glass. To confirm that a major part of the residual compressive stress is due to the mismatch in thermal expansion coefficients, the biaxial in-plane thermal stress σ(T) of the films were calculated, using the expression given in eq. (2):


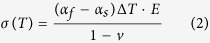


where α_f_ (1.0 × 10^−6^ °C) and α_s_ (given in Table 1) are the coefficients of linear thermal expansion of the film and the substrate, respectively, Δ*T* is the temperature difference between the deposition temperature and room temperature, and *E* and *v* are the Young’s modulus (800 GPa) and Poisson ratio (0.2) of the B:NCD film respectively^18^,^19^. The calculated thermal stress in the film is −(1.1 ± 0.1) GPa and −(1.9 ± 0.2) GPa for CE2000 and S-AF45, respectively, indicating that the compressive (σ(T) < 0) stress in the B:NCD film on S-AF45 glass is about 60% higher than on CE2000 glass.

For 2 membranes with the same etching diameter, higher compressive stress (induced by the underlying substrate) leads to a larger diameter and membrane area, when the membrane is formed upon release of the substrate. This higher compressive stress leads to a lower sensitivity since during pressure application the induced tensile stress has to overcome the compressive stress. For instance for 2 membranes of 550 μm etching diameter, the increase in diameter is about 0.4 μm larger on S-AF45 than on CE2000 with a correspondingly larger increase in surface area of about 330 μm^2^ larger for S-AF45 compared to CE2000, which gives rise to a higher sensitivity on CE2000 glass. A side-effect of membrane formation related to this increase in surface area is the appearance of wrinkles, i.e. out-of-plane-deformations as the NCD membrane is confined to the (relatively smaller) rim of the glass substrate (which is described in more detail in the section ‘Laser microscopy analysis’). In order to produce stress free, highly sensitive diamond layers for commercial applications an effort should be made to develop glasses that match the thermal expansion coefficient of diamond. In the development of lithium aluminosilicate glasses similar progress has been made already where glasses with tuneable viscosity and thermal expansion based on lithium oxide content were produced^20^. Nevertheless, the lowest thermal expansion coefficient reported was about 4 × 10^−6^ °C^−1^. To our current knowledge, CE2000 glass is the best available borosilicate glass. Glasses with extremely low thermal expansion coefficients (e.g. ZERODUR^®^) exist as well, but such low coefficients would cause tensile stress that might leads to delamination. In addition, it is important to note that non-uniform stresses, e.g. uniaxial tensile stress, can also lead to wrinkling^21^.

### Dopant-dependence of the sensitivity

**Figure 3:**
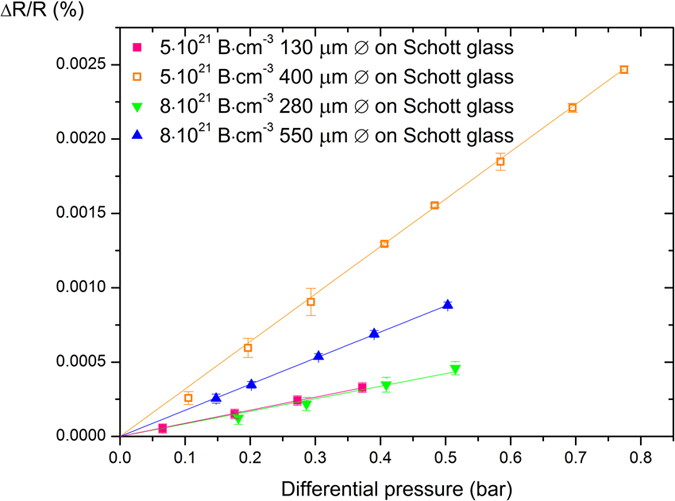
Relative change in resistance as a function of applied differential pressure for 4 membranes with 2 different doping levels and different sizes. The relative change in resistance increases with increasing membrane size and decreasing doping level.

In order to study the influence of the dopant concentration, 2 additional membranes were fabricated on S-AF45 glass, with diameters of 135 and 400 μm and an estimated boron concentration of 5·10^21 ^cm^−3^, as determined from Raman spectroscopy (Fig. 3) 22. The boron incorporation can be influenced by lowering the methane concentration, which leads to lower growth rates at the same temperature, inducing less structural defects within the grains and grain boundaries which have been shown to act as sites for local enhancement of B incorporation^13^,^14^. Here, a decrease in boron content was induced by an increased growth speed at slightly higher temperature^23^. Demlow *et al*. showed that for boron doping of diamond single crystals the incorporation rate of boron is not as fast as the growth rate at higher temperature, which means faster growth leads to lower boron concentrations^23^. In this work a corresponding increase in the sensitivity of the membranes is observed with a decrease in boron content. In previous works, a similar effect has been observed for the piezoresistivity of both boron doped diamond films and silicon pressure sensors^24^,^25^. The relative change in resistance is higher for lower doped materials, and although the absolute resistance increases and might complicate the measurement, this effect is relatively small for the small decrease in dopant concentration and can be considered negligible for measurement purposes^14^.

### Size-dependence of the sensitivity

The dimensions of the membranes also influence their sensitivity; the thinner the membrane the higher its sensitivity^26^. Although the growth of pin-hole free diamond films of about 50 nm has been reported^27^, membranes thinner than 150 nm are too fragile for the fabrication method used in this work and possibly also for the used pressure range^11^.

**Figure 4:**
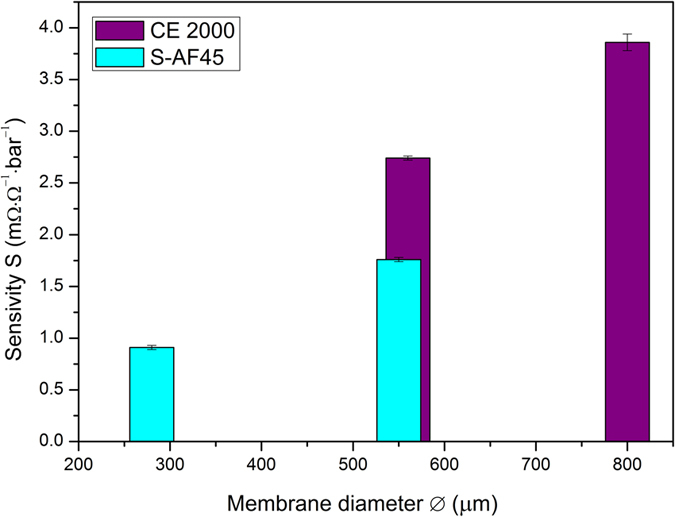
Pressure sensitivity (S) in function of membrane diameter. The standard error was calculated by averaging various measurement points (120 points, i.e. 2 min and a measurement each second) at a certain inflation pressure.

The relative change in resistance depends on the radius of the membranes as shown in Fig. 2. With an increase in the diameter of the membrane, there is a corresponding increase in sensitivity (Fig. 4). Going from a membrane diameter of 280 μm to 550 μm on S-AF45 glass the sensitivity is about doubled from (0.91 ± 0.02) mΩ·Ω^−1^·bar^−1^ to (1.76 ± 0.02) mΩ·Ω^−1^·bar^−1^. Similarly the sensitivity increases from (2.74 ± 0.02) mΩ·Ω^−1^·bar^−1^ to (3.86 ± 0.08) mΩ·Ω^−1^·bar^−1^ for increase in diameter from 560 μm to 800 μm on CE 2000 glass. In case of higher boron-doping level on S-AF45 glass, going from a membrane diameter of 130 μm to 400 μm on S-AF45 glass the sensitivity is increased from (0.87 ± 0.06) mΩ·Ω^−1^·bar^−1^ to (3.19 ± 0.02) mΩ·Ω^−1^·bar^−1^. The larger the membrane, the bigger the relative change in resistance and the more sensitive the membrane is. Possibly because of a larger amount of grain boundaries, which have been shown to be of primary importance for the piezoresistive behaviour of NCD membranes^12^. We observed experimentally, however, that an increase in membrane radius complicates the fabrication process, because bigger membranes tend to tear more easily since they have to carry a higher amount of etching fluid. For comparison, commercially available piezoresistive silicon pressure sensors have reported sensitivities around 10 mΩ·Ω^−1^·bar^−1^ for diaphragms with a size of 2 mm × 2 mm × 15 μm^28^. Although this value is still higher, as explained in the introduction, diamond can sustain harsh environments.

For non-wrinkled membranes the gauge factor can be written as


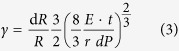


with *t* the membrane thickness, as shown in previous work^12^. If equation (1) is inserted into (3)


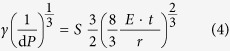


and the following is derived:


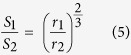


For wrinkled membranes, however, experimentally a power >2/3, and close to 1, is observed. With increasing membrane radius, the wrinkled area becomes relatively smaller compared to the central part of the membrane and thus we expect a change in dependence of the sensitivity on the membrane radius. In previous work it was shown that the resistance is independent of the size of the membrane by approximating a membrane with an infinitesimal amount of rectangular prisms with length *l(y)* and width *d(y),* with y the distance from the prism to the center of the membrane^11^. The change in resistance of a membrane upon inflation however does depend on the size of the membranes. For each prism the following equation holds:


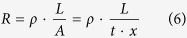


with *R*, the resistance, *L* the length, *A* the cross-sectional area and *x* the width of the rectangular prism and t and ρ the thickness and intrinsic resistivity of the diamond layer, respectively.

Assuming that ρ is constant and that the change in length is a manifold of the change in cross-sectional area *A*, i.e. *A* is considered as a constant and the following can be written


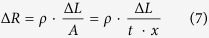



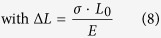


with, *σ* the induced stress, *E* the Young’s modulus and *L*_*0*_, the initial length of the rectangular prism. Upon straining of the membrane,


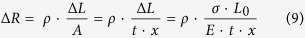


follows, which explains the dependence of the change in resistivity on the diameter (*L*_*0*_) of the membranes and relates it to the tensile stress induced upon straining under the assumption.

Nevertheless, the membrane radius is not the only determining factor, since wrinkled membranes have more degrees of freedom, their Young’s modulus might be lower and Poisson ratio higher, having an impact on their internal stress and thus sensitivity^29^. To gain more insight into the influence of increasing membrane size, flat, non-wrinkled membranes should be evaluated.

### Characterization

#### Scanning electron microscopy and Raman analysis

**Figure 5:**
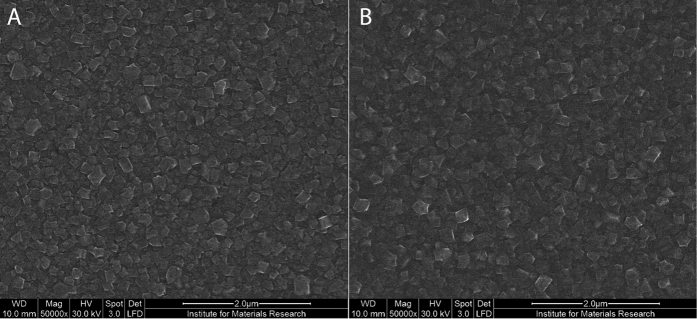
SEM images of 200 nm thick B:NCD films on (**A**) CE2000 glass and (**B**) S-AF45 glass.

In order to analyse the fabricated membranes more thoroughly and clarify the wrinkling patterns, further characterization was conducted. To ensure the deposited layer consists of diamond and to check the diamond quality SEM and Raman characterization was performed. The nanocrystalline structure of the deposited layers is analysed with SEM. Surface analysis of the 200 nm thick B:NCD films, confirms the presence of a closed well-faceted diamond layer with evidence of triangular facets of {111} octahedral grains and square {100} grains. The average grain size is estimated to be around 180 ± 10 nm (Fig. 5).

To confirm the deposited layers consists of diamond and to estimate their dopant level and quality, Raman spectroscopy was performed. The spectra show the presence of a diamond phase by the presence of the diamond optical phonon line (T_2g_ zone center mode) at 1332 cm^−1^ 30. Moreover, the deconvoluted Lorentzian component of the broad band at 500 cm^−1^ confirms the presence of boron dopant atoms above a concentration of 10^20 ^cm^−3^ 22. The diamond quality is often determined from the ratio between the diamond peak intensity and the peak intensity of the graphitic phase at 1550 cm^−1^ 31. Using the formula proposed by Silva *et al*., the estimated graphitic phase in the diamond membranes is 2–3%. The exact location of the diamond Raman peak is known to depend on the stress in the diamond layer. The diamond peak shifts to higher wavenumber under compressive stress and to lower wavenumber under tensile stress^30^. Therefore Raman spectroscopy is used to study the difference in stress in the NCD film before and after membrane formation. However, in order to do a qualitative comparison the influence of the doping level on the peak shift has to be eliminated. For boron doping, the diamond peak shifts towards lower wavenumber with increasing boron concentration, which is attributed to a combination of the self-energy shift due to the Fano interaction and tensile stress in the lattice from the incorporation of substantial quantities of boron^32^. To avoid distortion of the diamond lattice caused by dopant atoms which can influence the shift of the diamond Raman peak, the location of the diamond peak was measured for intrinsic NCD samples (grown under similar conditions as the B:NCD films from which the membranes were made but in absence of boron) (Table 1). For comparison, other factors that can influence the stress in the NCD film, e.g. growth conditions and grain size, are neglected as all samples were prepared and grown under identical conditions.

Based on the results, the dependence of the peak shift on the difference in thermal expansion coefficients does not seem to be linear. The small difference in peak position between NCD on CE2000 glass and NCD on silicon could be explained by the small difference in thermal expansion coefficient, i.e. 0.6 × 10^−6^ °C^−1^ (Table 1). In previous experiments it was observed that this difference in thermal expansion coefficient between NCD and fused silica causes tensile stress and in turn, possibly delamination of NCD layers thicker than 200 nm, as the thermal expansion coefficient of fused silica (0.5 × 10^−6^ °C^−1^) is lower than that of diamond^11^. For glass, however, the thermal expansion coefficient is higher than that of diamond causing compressive stress in the NCD layer, and delamination of the diamond layer from the substrate surface was not observed to occur. The biggest shift towards higher wavenumber is present for NCD films on S-AF45 glass confirming that these samples have the most compressive stress.

**Figure 6:**
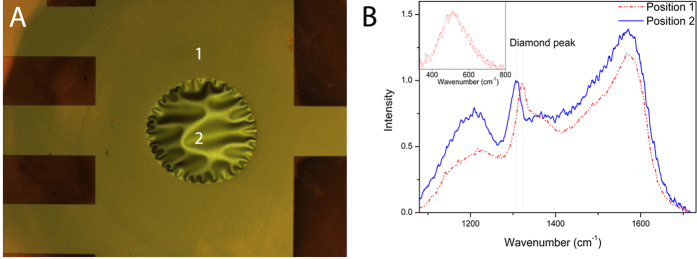
(**A**) Optical image of an NCD membrane on CE glass with 560 μm diameter. (**B**) Raman spectrum of the NCD film attached to the CE glass substrate (Position 1) and released to form a membrane (Position 2). The spectra shown are representative spectra, for each position (1 and 2) measurements were done at least at 3 different spots.

For the B:NCD films used in this work, the Raman diamond peak position lies around 1320 cm^−1^ compared to the more common value of 1332 cm^−1^. In order to explain the low peak position it is important to note that there are two contributions to the total stress, which are the growth-induced stress and the thermal stress^33^. The thermal stress is attributed to the difference in thermal expansion coefficient between the glass substrates and the diamond film. This effect will be discussed in the last section. The growth stress arises from defects and impurities, therefore the peak position at low wavenumber is attributed to boron doping, as was reported by Wang *et al*.^32^ Another possible explanation for peak positions at wavenumbers lower than 1332 cm^−1^ could be the grain boundary relaxation model, i.e. attractive interatomic forces across the grain boundaries induce stress in the grains due to a constrained relaxation^34^. In order to assess whether or not stress is released upon membrane formation because restrictions imposed by the underlying substrate are no longer present, the difference in the position of the diamond Raman peaks for B:NCD films still attached to the glass substrate (position 1) and those released as a membrane (position 2) was evaluated (Fig. 6). The diamond Raman peak shifts to lower wavenumber (2.8 ± 0.8 cm^−1^) going from position 1 (NCD film attached to the glass substrate) to position 2 (membrane). This shift is similar to the difference in peak shift between single crystal diamond and NCD on CE2000 glass mentioned in Table 1 and confirms (1.6 ± 0.5) GPa compressive stress, within the diamond film imposed by the substrate is released upon detachment from the substrate^35^.

**Figure 7:**
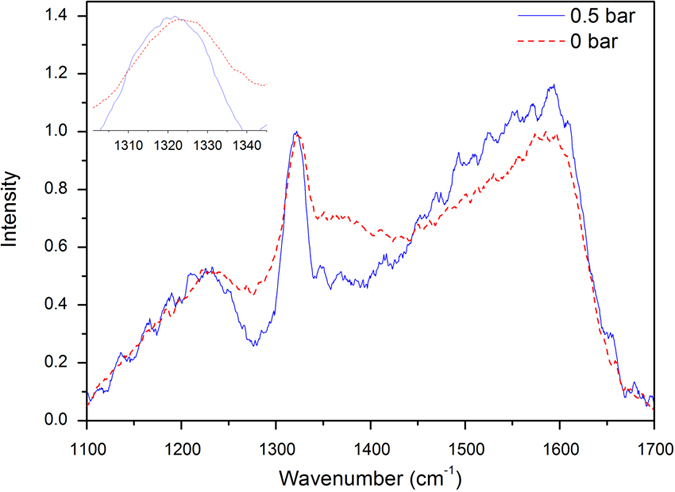
Raman spectra of a B:NCD membrane on CE2000 glass at a differential pressure of 0.0 bar (red dotted) and at 0.5 bar (blue solid) differential pressure. The spectra were measured in the center of the membranes, where the strain is the lowest and the smallest shift is expected to be measured. The inset shows the peak shift towards lower wavenumber under tensile stress caused by the inflation. The spectra shown are representative spectra, measurements both for inflated and non-inflated membranes were done at least at 3 different spots.

To compare the stress of a non-inflated with that of an inflated membrane, their respective diamond Raman peak locations were analysed for a membrane on CE2000 glass (Fig. 7). The peak shifted from (1322.5 ± 0.4) cm^−1^ to (1320.9 ± 0.6) cm^−1^ when the differential pressure changed from 0 to 0.5 mbar, i.e. a shift to lower frequency of nearly 2 cm^−1^, indicating tensile stress upon inflation. The tensile stress in the film caused by inflation is estimated to be (0.9 ± 0.3) GPa based on the peak shift^36^, this value has to be considered with care however, since this calculation doesn’t take the presence of wrinkles into account.

#### Laser microscopy analysis

**Figure 8:**
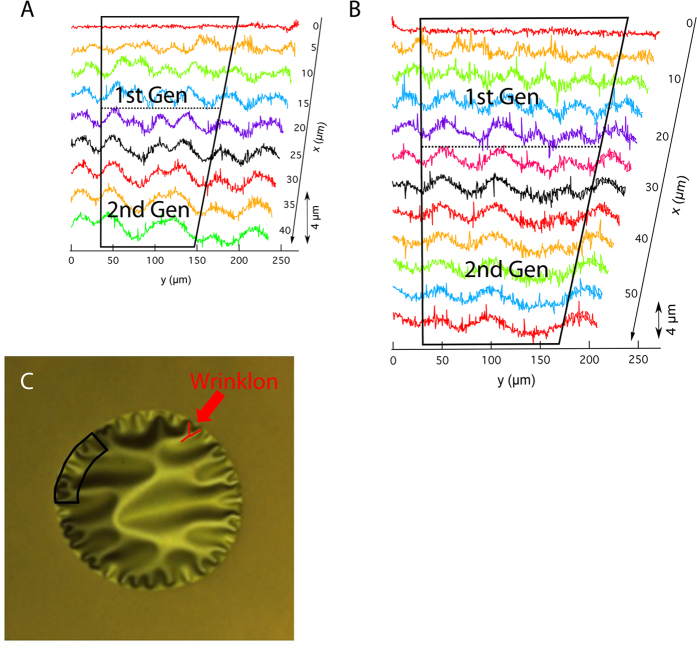
Topographic line profiles of the membranes of 550 μm on S-AF45 glass (**A**) and of 560 μm on CE2000 glass (**B**). The transition of the first generation wrinkles into the second, i.e. the so-called wrinklon (red Y-shape in (**C**)), takes place between the dotted line and the bottom black line in (**A**,**B**). The dotted line indicates the maximum amplitude for the maximum number of wrinkles, while at the lower black line only half of the original number of wrinkles are present. x = 0 is the edge of the membrane, x > 0 is in the direction of the center of the membrane. y follows the rim of the membrane, and was converted into a straight line. The black trapezoid is the analysed area of the membrane as shown in (**C**). The height profile, i.e. amplitude in z-direction, is shown by the double arrow indicating 4 micrometer.

With the aid of a laser microscope, two membranes of comparable size, i.e. 550 μm diameter and 560 μm diameter on S-AF45 and CE 2000 glass were used to compare the extent of wrinkling of membranes on the different types of glass, i.e. to evaluate the influence of stress on the wrinkled area (Fig. 8A,B). Vandeparre *et al*. showed that thin sheets under boundary confinement, such as a clamped membrane, spontaneously generate a universal self-similar hierarchy of wrinkles regardless of the material of the thin sheet^37^. The authors described wrinkled patterns by the appearance of wrinklons, a localized transition zone in the merging of two wrinkles (Fig. 8C). A wrinklon is basically the transition of 2 wrinkles close to the boundary or edge into 1 wrinkle at a further distance. The boundary condition in the case of our NCD membranes is the requirement that the membranes remain flat at the edges, while in the center they are allowed to relax which causes wrinkle and thus wrinklon formation. Argon *et al*. have reported similar wrinkles and wrinklons to lead to a hierarchical pattern for the blistering of thin films adhering on a thick substrate due to compressive stress^38^. Similar patterns have also been observed for the detachment of amorphous silicon films from SiO_2_/Si wafers^39^. Vella *et al*. suggested that for membranes wrinkling would occur in case of compressive stress, i.e. the membrane radius is confined to a smaller hoop^40^. The out-of-plane relaxation of NCD membranes into wrinkled patterns as observed upon membrane formation is most likely due to the residual compressive stress in the layer^41^. In case of substantial residual compressive stress, the membranes start wrinkling. Otherwise the fabricated membrane remains flat, which would happen in case there is no or a small difference in thermal expansion coefficient between the substrate and the NCD layer and no significant growth stress is induced. A higher thermal expansion coefficient for S-AF45 glass compared to CE2000 glass, explains a higher number of wrinkles for the films on S-AF45 glass. As a first comparison the number of wrinkles along the circumference of the membrane was counted. Indeed the density of the wrinkles is higher, i.e. the wavelength of the wrinkles is shorter, for the membranes on S-AF45 glass. On average the wavelength of the wrinkles is 33 μm for membranes on S-AF45 glass and 42 μm for membranes on CE2000 glass. Yan *et al*. predicted a similar effect, i.e. an increase in the number of wrinkles when the Young’s modulus of the surrounding film becomes increasingly larger compared to that of the central membrane^42^. Moreover, laser interferometry experiments demonstrate the emergence of first generation wrinkles, which are the wrinkles appearing at the rim, by clearly showing out-of-plane deformations. The height of these deformations is indicated by the double arrow in Fig. 8C. Furthermore, merging of the first generation wrinkles into the second by the formation of wrinklons is indicated by the dotted lines (Fig. 8A,B).

We found that this wrinklon formation, i.e. transition of first into second generation wrinkles, takes place closer to the edge for the membranes on S-AF45 compared to those on CE2000 glass, which means that both the wrinkles and wrinklons are shorter on S-AF45 substrates. Vandeparre *et al*. derived that tensile stress along the x-direction (cfr. x-direction of Fig. 8) increases the length of the wrinklons^37^. Based on this we postulate that higher residual compressive stress in the membranes, as determined by Raman, leads to shorter wavelengths of the wrinkles and wrinklons in their respective NCD membranes.

#### Temperature dependence of the wrinkles

**Figure 9:**
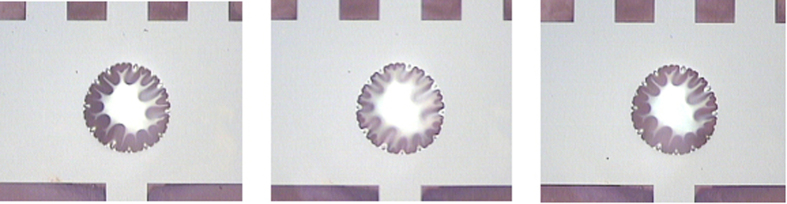
Optical image of the membrane of 560 μm in diameter on CE 2000 glass at room temperature (left), at 400 °C (center) and at room temperature after cooling (right). After cooling the membrane retains its original wrinkling pattern.

As shown in Fig. 9, the wrinkles are less pronounced at 400 °C, i.e. closer to the diamond deposition temperature, and retain their original form after cooling again, which indicates that the B:NCD films are thermally stressed.

As stated before, more wrinkles appear in more compressed membranes. In order to verify, the number of wrinkles of a membrane 560 μm in diameter on CE2000 was compared at room temperature and at 400 °C (Fig. 9). There is no definite decrease in the number of wrinkles. However, as seen in the optical micrographs, the wrinkles are less individually resolved at room temperature due to the resolution of the images and light scattering of the membrane. At elevated temperature the membrane appears to be more flat, i.e. the incoming light is reflected more at the edges compared to at room temperature, which indicates less compressive stress. Lower induced compressive stress at elevated temperatures can be explained by the linear expansion of the glass substrate in every direction, leading to an increase in hole diameter of about 0.1% and thereby reducing the compressive stress by 0.3 ± 0.1 GPa onto the diamond membrane as the B:NCD film does not expand to the same extent as the glass substrate^43^.

## Conclusion

In conclusion, the optimization of a robust and versatile pressure sensor, resistant to a variety of media in which commercially available silicon sensors don’t survive, is reported. As differential pressure is measured, the sensor can be used in a wide pressure range with a maximum absolute pressure difference of 0.8 bar. The sensitivity of the B:NCD membranes fabricated for this work is related to the residual stress, which contains both growth and thermal stress, in the diamond film as deduced from the Raman peak location on B:NCD and undoped NCD membranes. The thermal stress of the film depends on the substrate material and more specifically on its thermal expansion coefficient which was confirmed by optical micrographs at room and elevated temperature and by the Raman diamond peak location for membranes on different substrates. For B:NCD on glass compressive stress is induced during growth leading to wrinkle formation induced by out-of-plane deformations which release stress upon removal of the substrate, as shown by laser scanning micrographs. The pressure sensitivity can be related to the amount of wrinkling, i.e. samples that are more wrinkled, because they were more compressed initially, need a bigger applied differential pressure for the same response as samples that are less wrinkled. Therefore the sensitivity is 55% higher for the membranes on CE2000 glass compared to membranes on S-AF45 glass because the films on CE2000 glass are less compressed due to a smaller difference in thermal expansion coefficient between this type of glass and the B:NCD film. These results show the potential of B:NCD films as very sensitive pressure sensors. The sensitivity can be enhanced easily by an increase of the dopant concentration and clever choice of the substrate material: a substrate with a thermal expansion coefficient as close as possible to that of diamond or ideally even smaller ensures flat B:NCD layers as long as delamination is not an issue. The sensitivity of these devices is expected to be the highest possible and the thermal expansion coefficient of the substrate material should be taken into account in the future design of B:NCD based pressure sensors.

## Methods

Schott AF45 (S-AF45) and Corning Eagle 2000 (CE2000) (1 cm × 1 cm × 710 μm) glass substrates were cleaned with standard RCA-1 and RCA-2 cleaning procedures^44^. The substrates were seeded with a water-based colloidal suspension of ultradispersed detonation diamond via drop casting and subsequent spin-drying^45^. The suspension of detonation nanodiamonds from NanoCarbon Institute Co., Ltd., features a zeta potential of (45 ± 5) mV and contains particles with a size of 6–7 nm. The growth of 200 nm thick B:NCD layers is performed in an ASTeX 6500 series microwave plasma enhanced chemical vapour deposition (MWPECVD) system using a plasma containing 3% methane and trimethylboron (TMB) in a TMB/methane ratio of 10 000 ppm in hydrogen. The film thickness was monitored by *in-situ* laser reflection interferometry. The microwave power and pressure were 2100 W and 11 Torr respectively to ensure a substrate temperature of 550 °C ± 50 °C, which is below the glass transition temperature of 663 °C and 722 °C for S-AF45 and CE2000 respectively. The substrate temperature was determined by a Cyclops 52, Minolta hand-held infrared pyrometer.

The fabrication of the membranes in the center of a B-NCD Hall bar structure is documented in previous work^11^. The fabricated membrane pressure sensors were loaded into a home-built measuring set-up that was designed to inflate membranes with positive differential pressures at ambient conditions. During inflation, four-point resistance measurements were performed with a Keithley 2400 multimeter by application of a fixed DC current. In order to eliminate temperature effects caused by the current flow, measurements were started only after the resistance reached a stable value.

The B:NCD layers were characterized with a FEI Quanta 200 FEG-SEM scanning electron microscope operated at 15 kV and with a Horiba Jobin Yvon T64000 Raman spectrometer equipped with a BXFM Olympus 9/128 microscope, a Horiba JY Symphony CCD detector and a 488 nm Lexell SHG laser, with a resolution of 0.64 cm^−1^. The Raman spectra were fitted using Fityk software with Lorentzian peaks to determine the intensity and peak locations. 3D surface images were taken with a Keyence VK-9700 laser microscope at a resolution of 0.69 um/pixel and a height resolution of 1 nm.

## Additional Information

**How to cite this article**: Drijkoningen, S. *et al*. The pressure sensitivity of wrinkled B-doped nanocrystalline diamond membranes. *Sci. Rep.* **6**, 35667; doi: 10.1038/srep35667 (2016).

## Figures and Tables

**Table 1:** Raman shift of the diamond peak in relation to the substrate.

**Substrate**	**Diamond peak position (cm**^**−1**^)	**Diamond peak shift wrt single crystal diamond**	**Thermal expansion coefficient (°C**^**−1**^)	**Estimated Induced stress (GPa)**
S-AF45 glass	1337.0 ± 0.5	5.3 ± 0.5	4.5 × 10^−6^ 46	3.0 ± 0.3
CE2000 glass	1333.8 ± 0.3	2.1 ± 0.4	3.2 × 10^−6^ 47	1.2 ± 0.2
Silicon	1333.9 ± 0.5	2.2 ± 0.5	2.6 × 10^−6^ 48	1.2 ± 0.3
Single Crystal Diamond	1331.7 ± 0.2 49	/	1.0 × 10^−6^ 50	/

The peak position for single crystal diamond is shown as a reference.
